# Tripolar concentric ring electrodes for capturing localised electroencephalography signals during sleep

**DOI:** 10.1111/jsr.14203

**Published:** 2024-03-27

**Authors:** Nicole Stuart, Jack Manners, Eva Kemps, Phuc Nguyen, Bastien Lechat, Peter Catcheside, Hannah Scott

**Affiliations:** ^1^ Flinders Health and Medical Research Institute: Sleep Health Flinders University Adelaide South Australia Australia; ^2^ College of Education, Psychology and Social Work Flinders University Adelaide South Australia Australia

**Keywords:** EEG acquisition, healthy sleepers, quantitative EEG, sleep stages, TCRE, tripolar concentric ring electrodes

## Abstract

By design, tripolar concentric ring electrodes (TCRE) provide more focal brain activity signals than conventional electroencephalography (EEG) electrodes placed further apart. This study compared spectral characteristics and rates of data loss to noisy epochs with TCRE versus conventional EEG signals recorded during sleep. A total of 20 healthy sleepers (12 females; mean [standard deviation] age 27.8 [9.6] years) underwent a 9‐h sleep study. Participants were set up for polysomnography recording with TCRE to assess brain activity from 18 sites and conventional electrodes for EEG, eyes, and muscle movement. A fast Fourier transform using multitaper‐based estimation was applied in 5‐s epochs to scored sleep. Odds ratios with Bonferroni‐adjusted 95% confidence intervals were calculated to determine the proportional differences in the number of noisy epochs between electrode types. Relative power was compared in frequency bands throughout sleep. Linear mixed models showed significant main effects of signal type (*p* < 0.001) and sleep stage (*p* < 0.001) on relative spectral power in each power band, with lower relative spectral power across all stages in TCRE versus EEG in alpha, beta, sigma, and theta activity, and greater delta power in all stages. Scalp topography plots showed distinct beta activation in the right parietal lobe with TCRE versus EEG. EEG showed higher rates of noisy epochs compared to TCRE (1.3% versus 0.8%, *p* < 0.001). TCRE signals showed marked differences in brain activity compared to EEG, consistent with more focal measurements and region‐specific differences during sleep. TCRE may be useful for evaluating regional differences in brain activity with reduced muscle artefact compared to conventional EEG.

## 1 INTRODUCTION

The ‘gold standard’ objective method of measuring sleep is polysomnography (PSG). This method uses electroencephalography (EEG) to record brain electrical activity, from which wake, rapid eye movement (REM) and non‐REM sleep are typically scored manually in 30‐s epochs. This approach provides a standardised method for evaluating sleep and has underpinned sleep medicine and the discovery of many sleep phenomena, including differentiating non‐REM and REM sleep, which is key for health focused PSG and cognitive focused PSG, as well as the presence of alpha waves that is used in marking sleep onset (Patra et al., 2022). However, there are several limitations for which alternative EEG measurement practices may have advantages.

With appropriate sample rates, conventional EEG recordings have high temporal, but poor spatial resolution. This is due to effects of distance, temporospatial summation and resistive layers between the brain and recording electrodes (Burle et al., 2015), resulting in a signal that is a combination of brain and muscle activity sources within a region of approximately 6–9 cm in diameter around paired electrodes (Babiloni et al., 2001). To estimate more localised brain activity, surface Laplacian transforms can be used to combine signals from an array of electrodes (typically between 64 and 256‐electrode EEG setup) to estimate more localised spatial sources of brain activity. This statistical approach requires high‐density electrode placements, high‐quality signals, and computational resources for the calculation of localised activity (Kayser & Tenke, 2015), where more electrodes enable better detection of localised activity (Jones et al., 2014; Wang et al., 2020). The required EEG setup for these transformations is impractical for most sleep recordings. Additionally, conventional EEG is often contaminated by cardiac and muscle artefacts (Janani et al., 2017), especially during events characterised by high muscle activity (e.g., wake, body movements, teeth‐grinding in sleep). These artefacts can mask brain activity and render epochs too noisy for reliable sleep analysis and scoring. Further, given that specific markers have been identified in localised brain activity, such as memory consolidation (Muehlroth et al., 2019), post‐traumatic stress disorder (Wang et al., 2020) and mortality (Lechat et al., 2022), traditional EEG measurement using conventional electrodes may mask other potentially useful underlying signal features during sleep. These limitations may be overcome with novel electrode technologies.

This study was designed to evaluate the novel use of tripolar concentric ring electrodes (TCRE) to assess sleep. By electrode design, TCRE record more localised brain activity underlying each electrode compared to more distantly spaced paired electrodes. This potentially has advantages of recording more localised activity with superior signal: noise compared to traditional EEG electrodes. TCREs consists of three elements: a central conducting disc and two electrically isolated surrounding rings. Signals from the outer and inner rings are referenced to the central ring to derive a focal differential signal that reflects current density and closely approximates the surface Laplacian potential from a single electrode without the need for a much larger array of more distantly spaced conventional electrodes (Besio et al., 2006). A further consequence is that TCRE signals exhibit superior signal: noise and are less impacted by electromyography (EMG) artefact compared to traditional EEG, which typically also records EMG activity from nearby muscles, especially during wake (Lei & Liao, 2017).

The TCRE has previously been used to identify markers of seizure during wake for patients with epilepsy (Besio et al., 2013; Besio et al., 2014; Makeyev et al., 2013), but has not previously been systematically tested for sleep assessments. Thus, this study sought to directly compare TCRE and more conventional EEG signals using quantitative EEG analytical approaches applied to sleep study recordings using TCRE electrodes and EEG derived from the outer‐rings of two distantly placed TCRE electrodes.

## 2 METHODS

### 2.1 Participants

Participant inclusion criteria were: age (18–65 years); self‐reported regular good sleep (habitual bedtime between 10:00 p.m. and 12:30 a.m.; habitual wake up time between 7:00 and 9:00 a.m.; total sleep time between 6 and 8 h); with an ‘intermediate chronotype’ according to the Morningness–Eveningness Questionnaire; and a body mass index (>18.5 and <32.9 kg/m^2^). Participants were excluded if they: had been diagnosed with a sleep disorder; reported symptoms suggestive of a sleep disorder; were taking any medications, drugs or substances known to affect sleep; had undertaken any trans‐meridian travel or shift work in the 2 months prior to the study; or were a current smoker. Ethics approval was received from the Flinders University Human Research Ethics Committee (project no: 4441). All participants provided written informed consent.

### 2.2 Design

A within‐subjects observational study design compared brain activity signals (TCRE, EEG) across conventionally scored sleep stages (wake, rapid eye movement [REM] sleep, non‐REM Stage 1 [N1], Stage 2 [N2], and Stage 3 [N3] sleep) during an overnight sleep opportunity. Participants arrived at the sleep laboratory ~6:00 p.m. and were set up for conventional overnight PSG with the addition of TCRE. Participants were then given a 9‐h sleep opportunity (10:00 p.m. until 7:00 a.m.), with sleep signals continuously monitored by laboratory staff to ensure adequate signal quality overnight.

### 2.3 Measures

**FIGURE 1:**
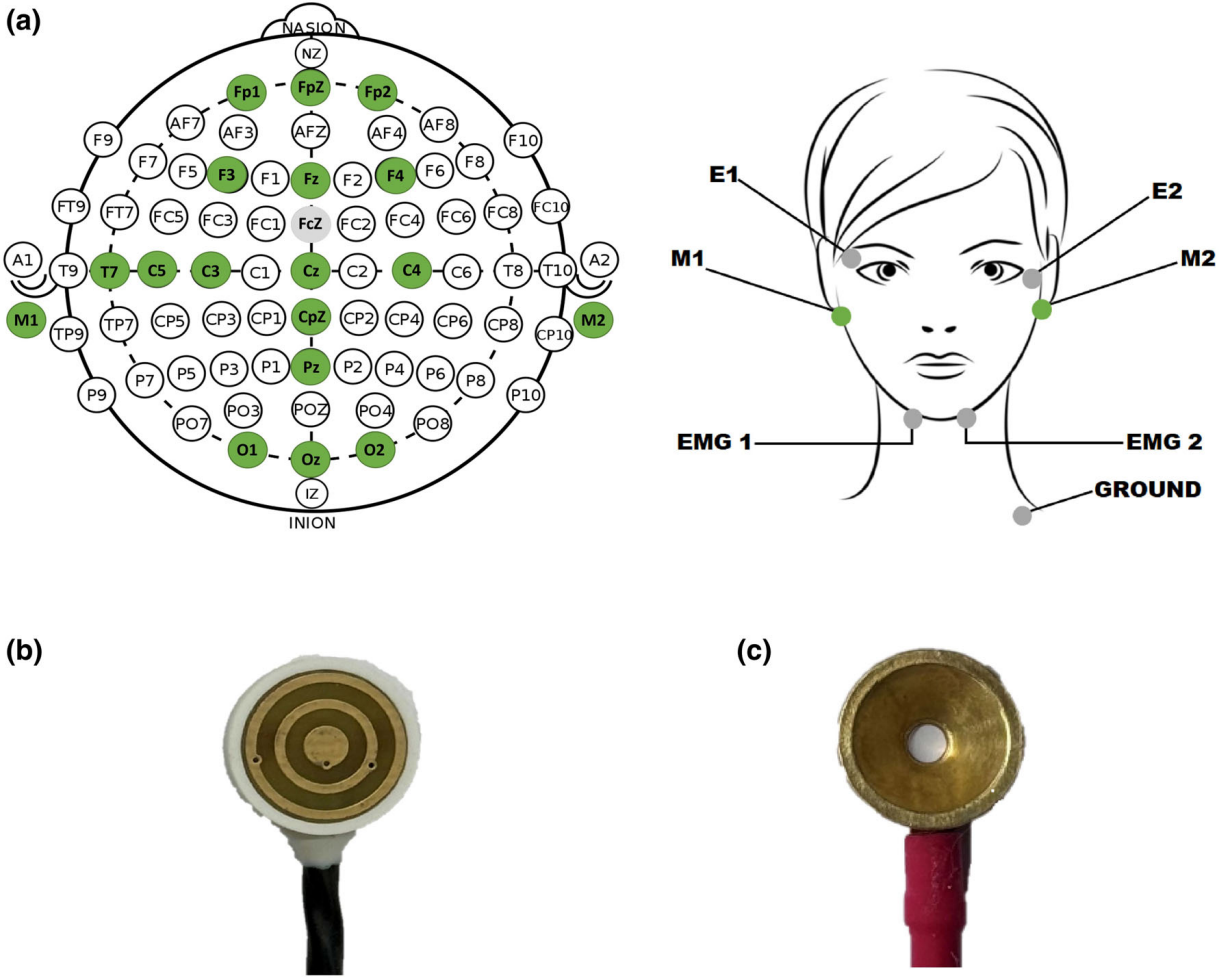
(a) Electrode positioning for tripolar concentric ring electrodes (TCRE) (green sites) and traditional electrodes (grey sites) using the 10–20 system. TCRE (b) compared to standard gold cup electrode (c).

**FIGURE 2:**
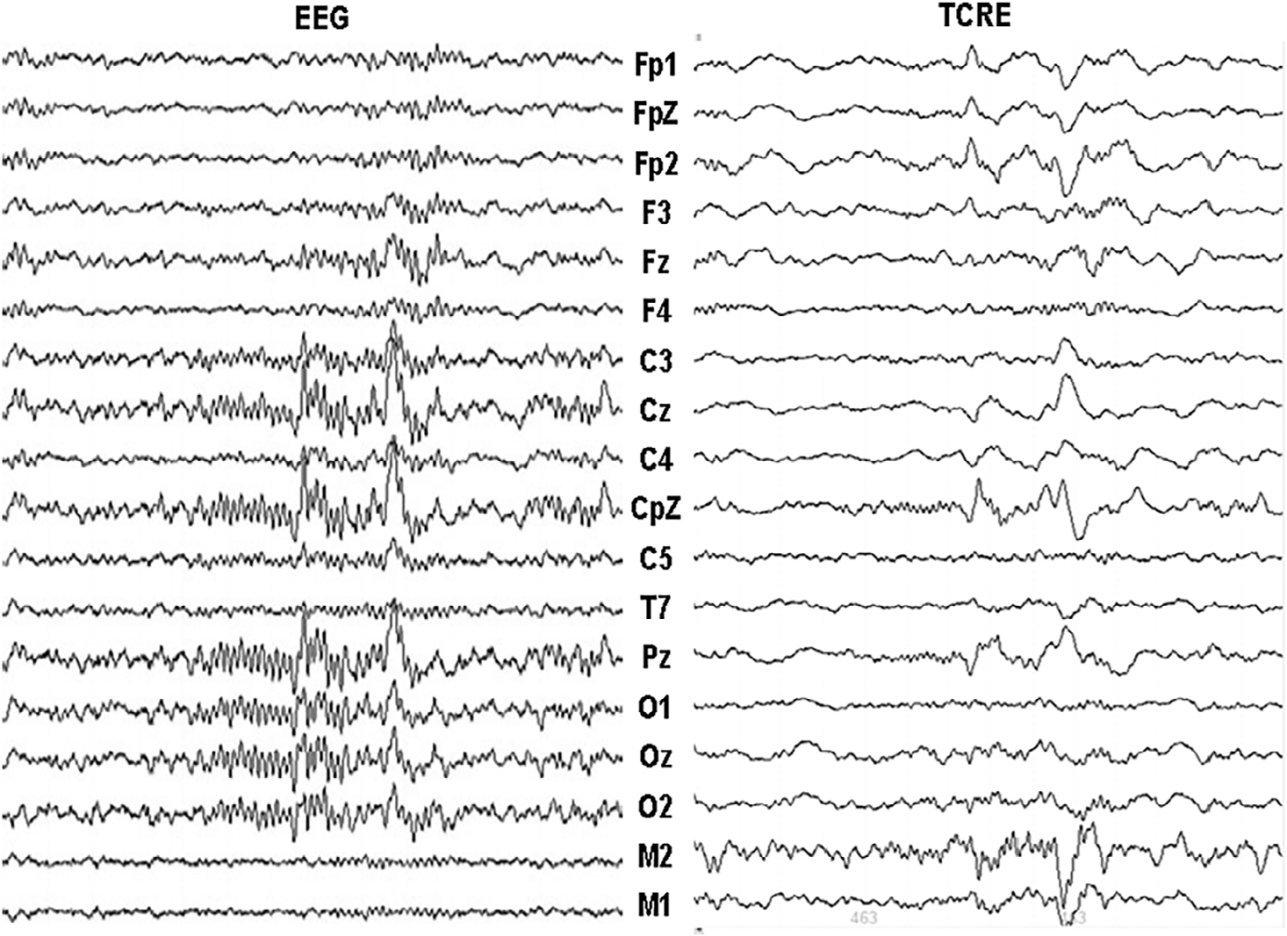
Electroencephalography (EEG) trace layout (left) and TCRE trace layout (right) during an epoch of N2 sleep. For EEG trace layout and TCRE trace layout for wake, N1, N3, REM, and marked arousals, see Data S1 Figures S1–S5. REM, rapid eye movement; TCRE, tripolar concentric ring electrodes.

The PSG recording was conducted to collect EEG, EMG, electro‐oculography (EOG), and electrocardiography (ECG) signals. The TCRE and conventional EEG signals were simultaneously recorded by connecting individual TCRE signal outputs and the outer rings of two TCRE, referenced according to conventional electrode configurations into a sleep data acquisition system (Compumedics Grael 4 K, Melbourne, Australia) and sampled at 512 Hz. Electrode placement followed the standard EEG 10–20 electrode placement system. In total, 18 TCRE were placed on the scalp, along with a further six EEG electrodes for EEG referencing (CFz, M1, M2), grounding, EMG, and EOG (see Figure 1 for electrode positions). An independent sleep scorer used the EMG, EOG and EEG signals derived from the outer rings of paired TCRE electrodes, electrically equivalent to conventional EEG, to score the recording in 30‐s epochs to sleep stages according to standard American Academy of Sleep Medicine (AASM) sleep scoring criteria (Berry et al., 2020; Figure 2). As the TCRE signals themselves substantially differ from conventional EEG, these sleep scoring criteria were not applied directly to the TCRE signals.

The PSG data were exported into European data format (EDF) for analysis along with scored sleep stage files using Compumedics Profusion software (version 4.0). A fast‐Fourier transform (FFT) was applied using multitaper‐based estimation, on non‐overlapping 5‐s epochs (for more information on the methods see Lechat et al., 2021). Absolute power in five frequency bands was calculated for each 5‐s epoch: (delta [0.5–4.5 Hz], theta [4.5–8 Hz], alpha [8–12 Hz], sigma [12–15 Hz], beta [15–32 Hz]): by computing the area‐under the curve of power spectrum density over the specified frequency range. Relative powers were then calculated by dividing the absolute power of a given frequency range by the sum of absolute powers for all frequency ranges. The resulting relative power ranges from 0% to 100%, and the sum of relative powers for all frequency bands for one epoch equals 100%. Average and maximum spectral power in each frequency band during each 5‐s epoch were calculated separately for the manually‐scored wake, N1, N2, N3 and REM sleep epochs.

### 2.4 Statistical analyses

Linear mixed‐effects models were conducted using the IBM Statistical Package for the Social Sciences (SPSS, version 27) to investigate the interactions between brain activity signals (TCRE versus EEG) and sleep stages on relative spectral power, separately for each frequency band (delta, theta, alpha, sigma, beta). Participant ID was specified as a random intercept to account for expected between‐individual variability. Normality of data split by each power spectral was assessed using Q–Q and distribution plots. For the most part, data were normally distributed, so untransformed data were retained to simplify interpretation. Statistical significance was considered as *p* < 0.05. Where appropriate, differences are reported with 95% confidence intervals (CIs). Estimated multi‐taper spectrograms were created from the Cz (TCRE) and Cz‐M1 (EEG) channels, using 30‐s windows spaced at 5‐s intervals.

A further exploratory analysis was conducted to help visualise localised brain activity using MNE‐python (version 1.4, Gramfort et al., 2013). Power spectral density (PSD) was computed for the sleep data to explore and compare PSD between EEG compared to TCRE at different recording sites.

Characterisation of noisy epochs was calculated for each TCRE and EEG electrode pair, using Python (version 3.10) and the ‘scikit‐learn’ package (version 1.1.1) based on the maximum power value found for each 5‐s epoch. To normalise skewed data, a log‐10 transformation was applied to both the TCRE and EEG data after the addition of a small constant of 0.01 to avoid zero values. Both datasets were then standardised by subtracting the median and dividing by the interquartile range (see robust scaler, Pedregosa et al., 2011). A cut‐off value corresponding to a maximum power of 400 μV^2^ in the 5‐s epoch was chosen to characterise noisy epochs in the EEG data, based on previous work (Sweetman et al., 2021). The equivalent TCRE cut‐off was then calculated based on the inverse transformation from the robust scaler to represent the corresponding threshold value in the scaled TCRE data. Following transformation and standardisation of 5‐s epoch data, an equivalent cut‐off for TCRE signals was derived as 8928 μV^2^. The cut‐offs were then used to derive the number of noisy epochs for each electrode type. Odds ratios with Bonferroni adjusted 95% CIs were calculated to determine the proportional differences in the number of noisy epochs between electrode types, with Fisher's exact estimates of significance.

## 3 RESULTS

### 3.1 Demographics

A total of 20 healthy participants (12 females, eight males) with a mean age around 30 years and with a self‐reported habitual sleep time of around 7 h/night completed the study (Table 1).

**TABLE 1:** Participant's demographics.

Variable	Mean (SD)	Range
Age, years	29.6 (11.6)	18–56
Body mass index, kg/m^2^	22.2 (3.6)	17.6–31.9
Habitual bedtime	10:45 p.m. (43 min)	9:30 p.m.–12:00 a.m.
Habitual wake time	7:22 a.m. (52 min)	6:00 a.m.–8:30 a.m.
Reported average total sleep time, h	7.6 (0.9)	6–9
Insomnia Severity Index score	2.3 (2.1)	0–7
Pittsburgh Sleep Quality Index score	3.3 (1.6)	0–7
Epworth Sleepiness Scale score	4.6 (3.5)	0–11

Abbreviation: SD, standard deviation.

### 3.2 Spectral power of TCRE and EEG signals

**FIGURE 3:**
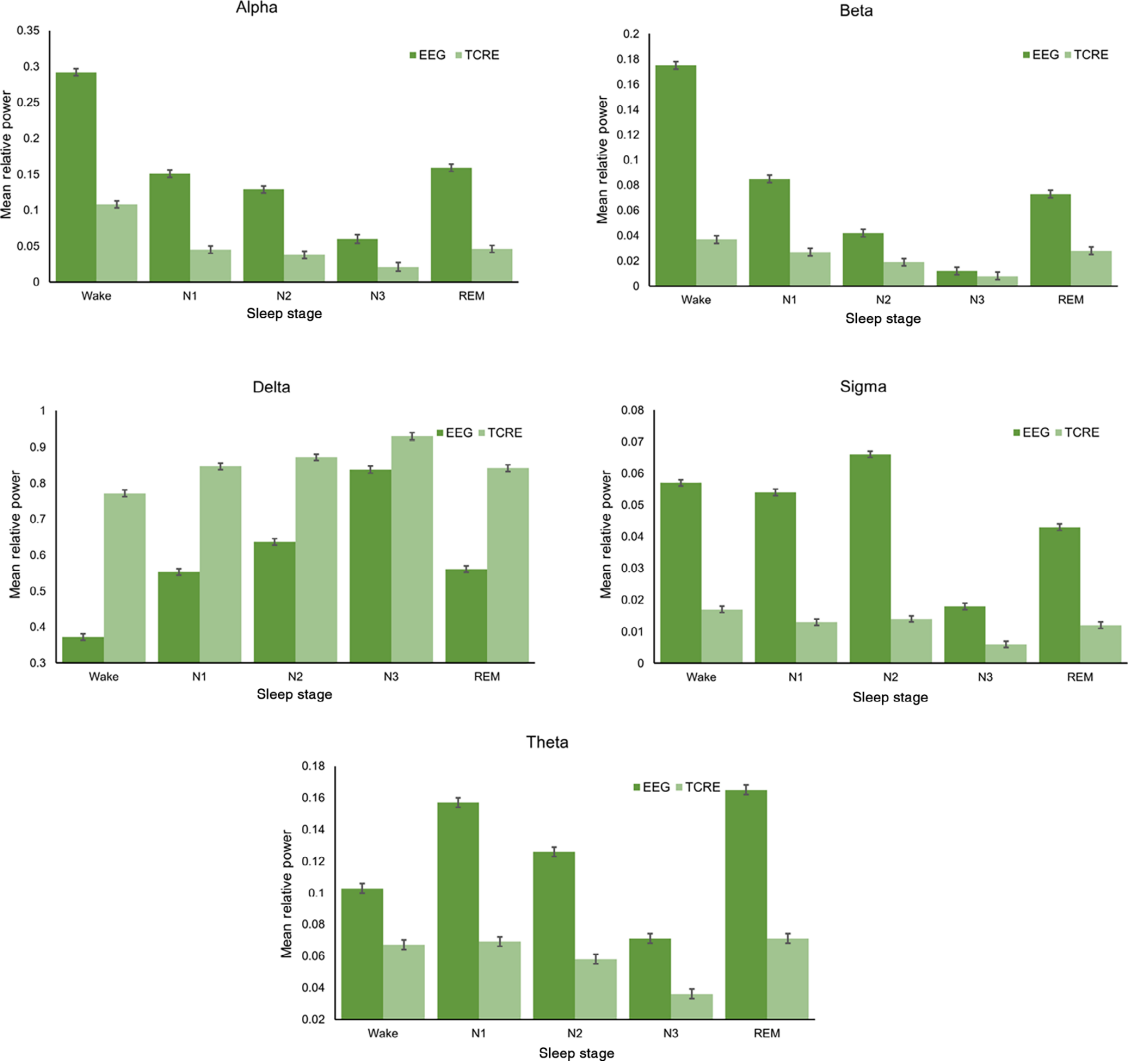
Mean relative spectral power by power band. Mean relative spectral power for each sleep stage is shown for each power band and for EEG and TCRE. Error bars show standard error. See Data S1 Table S1. EEG, electroencephalography; REM, rapid eye movement; TCRE, tripolar concentric ring electrodes.

**FIGURE 4:**
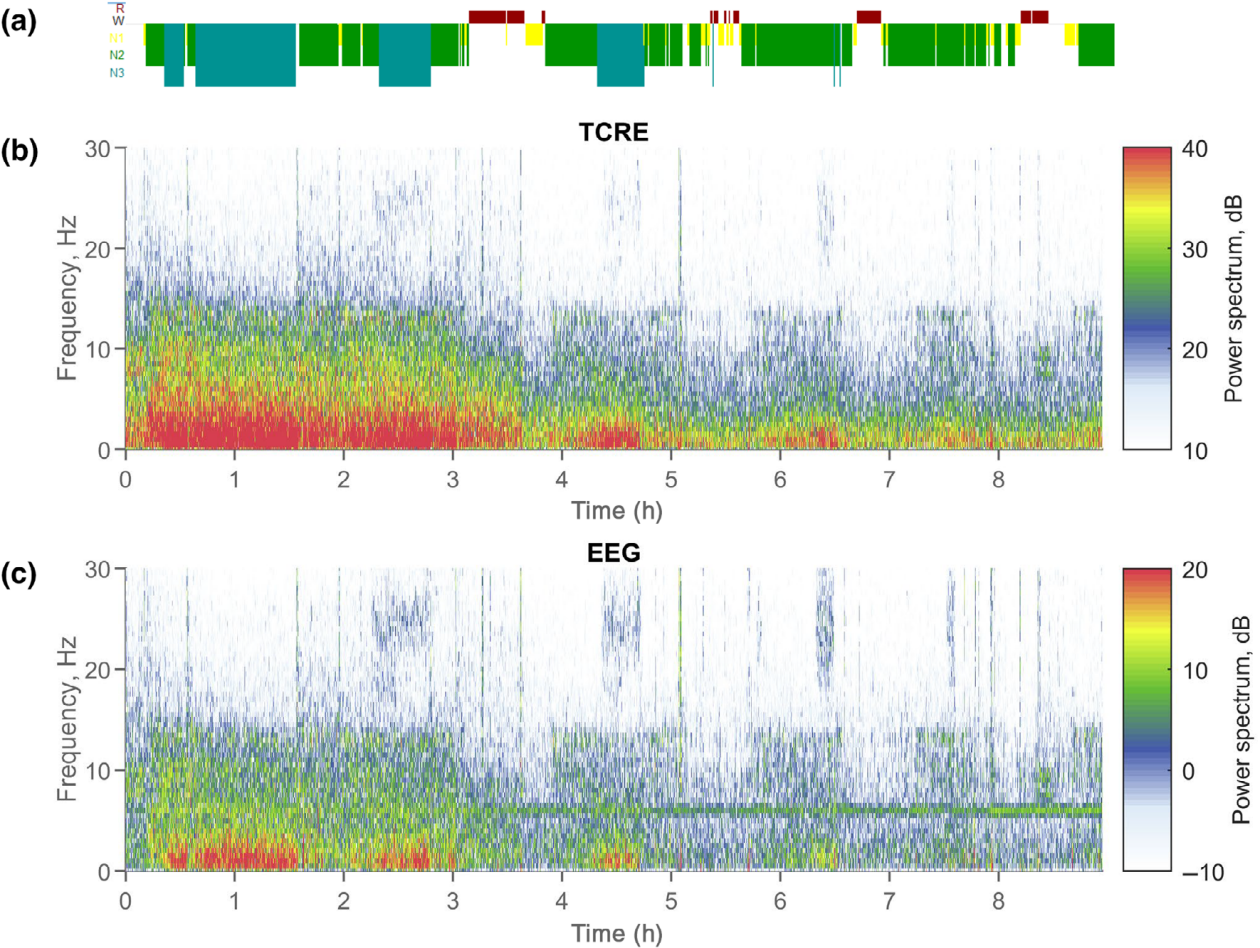
Whole night hypnogram (a) and frequency spectrograms (b, TCRE; c, EEG) for one participant. An example whole night recording from one participant showing the conventional hypnogram obtained from manual scoring based on American Academy of Sleep Medicine recommended sleep staging (a), and time (h) versus frequency (Hz) spectrograms derived from TCRE (b, from Cz) and EEG (c, from Cz‐M1) signals, with colours indicative of signal power, calculated as decibel (dB) values using the reference signal of 1 μV. EEG, electroencephalography; TCRE, tripolar concentric ring electrodes.

Figure 3 summarises the overall relative spectral power in TCRE versus EEG within each frequency band across the group data, and Figure 4 shows an example from one participant to show the conventional hypnogram and EEG versus TCRE spectrograms across a whole night recording. Linear mixed models demonstrated significant interaction and main effects of both signal type and sleep stage (all *p* < 0.001) on relative spectral power in each power band. Pairwise comparisons showed that TCRE relative spectral power was lower in all sleep stages for alpha, beta, sigma, and theta power than EEG across all sleep stages (*p* < 0.001), except there was no significant difference in beta relative power during N3 sleep (*p* = 0.199). For delta, relative spectral power was higher in all stages with TCRE compared to EEG (*p* < 0.001) (Figure 3).

### 3.3 Spatial differences

**FIGURE 5:**
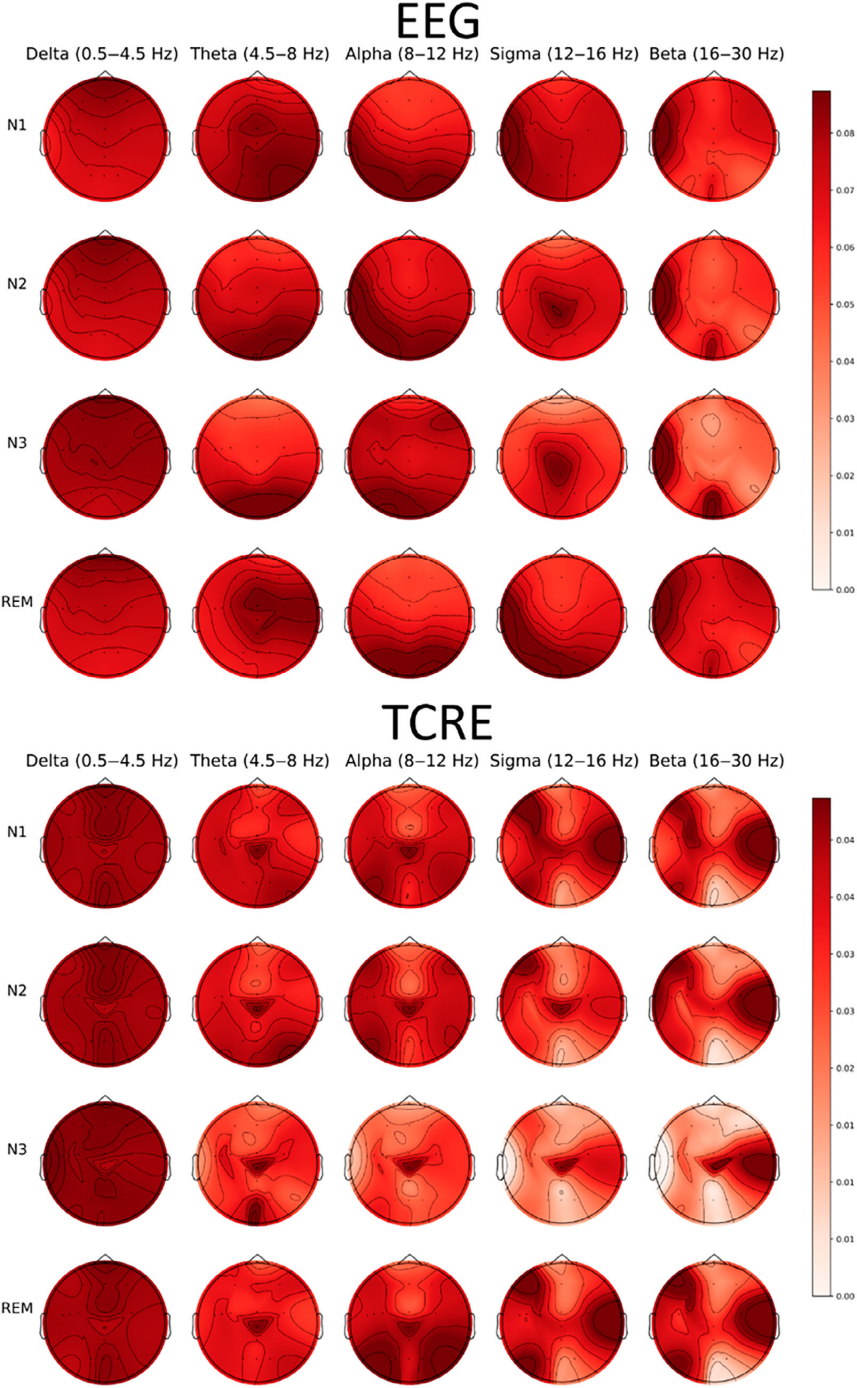
Overall relative spectral power of EEG versus TCRE across sleep stages. Scalp topography, based on relative power from all sites recorded (see Figure 1), indicating activation of signals across five frequency bands (delta: 0.5–4.5 Hz, theta: 4.5–8 Hz, alpha: 8–12 Hz, sigma: 12–15 Hz, and beta: 15–32 Hz) associated with different sleep stages. The darker the red, the higher the activation in that area. EEG, electroencephalography; TCRE, tripolar concentric ring electrodes.

Relative signal power was differentially localised in EEG versus TCRE measurements. Figure 5 shows the combined differential spatial localisation of relative brain power derived from EEG versus TCRE across sleep stages in all participants. Despite differences in the location of power distribution, all other power bands were far more localised in the TCRE recordings than the EEG counterparts. For example, beta power appeared more localised to the right parietal lobe through all sleep stages, and alpha activity during REM was far more prominent in occipital than frontal regions.

### 3.4 Noisy epochs

**FIGURE 6:**
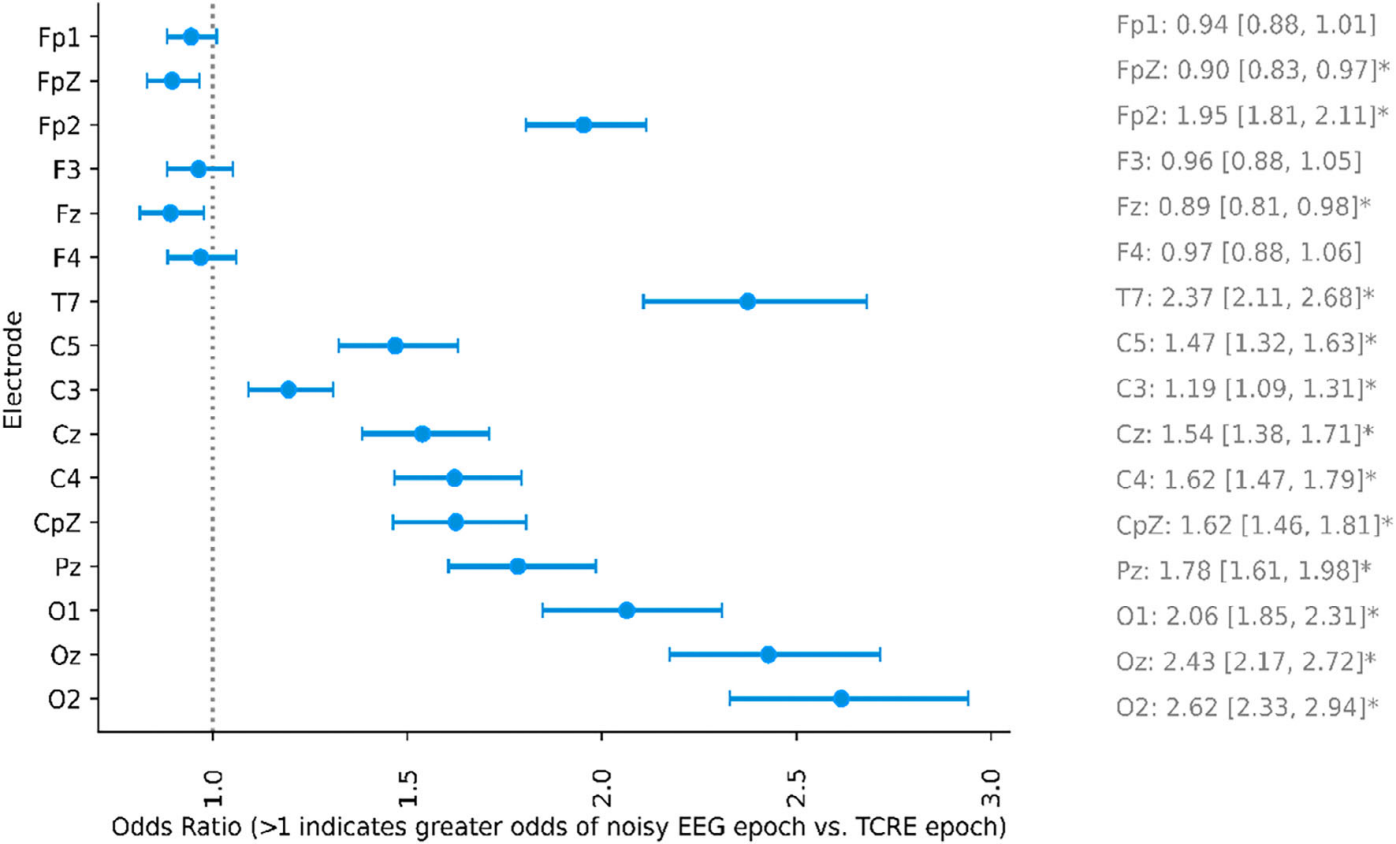
Odds ratio of EEG versus TCRE epochs being classified as ‘noisy’ for each electrode site. *Bonferroni‐adjusted Fisher‐exact *p* < 0.05. EEG, electroencephalography; TCRE, tripolar concentric ring electrodes.

At the central, parietal, and occipital electrode locations, EEG showed an approximately twofold increase in the number of noisy epochs compared to TCRE (Figure 6). There were no differences in the number of noisy epochs in most frontal electrode locations, except for FpZ and Fz, where TCRE showed slightly more noisy epochs than EEG.

## 4 DISCUSSION

This study compared the brain activity signals derived from TCRE to more traditional EEG during a night‐time sleep opportunity in healthy volunteers. TCRE exhibited higher relative power in the delta frequencies and lower power in the alpha, theta, sigma, and beta frequencies than EEG. Differences in activity across brain regions, displayed via topographical plots, are suggestive of more focal acquisition of brain activity. Furthermore, although noisy epochs were relatively infrequent with both signal types, TCRE had less noisy epochs than EEG in central, parietal, and occipital locations. These features support the potential utility of TCRE as a valuable modality for capturing more localised brain activity during sleep than can be captured by traditional EEG alone.

The TCRE showed marked differences in relative spectral power across sleep stages compared with EEG. TCRE signals contained less relative beta power than EEG, consistent with less muscle artefact infiltration in wake, N1 and N2 sleep, given that power in the beta frequency range is thought to predominantly originate from muscle activity (Kang et al., 2020). However, there was no difference between TCRE and EEG in relative beta power during N3 sleep, which is perhaps unsurprising given the predominance of delta and markedly reduced beta power during N3 sleep (Benbadis, 2006). Relative delta power was higher for TCRE signals compared to EEG signals across all sleep stages, potentially also suggesting less muscle artefact intrusion.

The TCRE signals showed clear temporospatial differences between recording sites, with higher activation and more localised activity in TCRE signals compared to EEG signals. This difference likely reflects much wider electrode spacing, and thus lower spatial resolution, with EEG. One notable difference was in the alpha frequency band across sleep stages. EEG remained relatively consistent in comparison to TCRE, which demonstrated more variable alpha, sigma, theta, and beta activation during NREM sleep, again consistent with higher spatial resolution and more localised differences in brain activity only discernible with TCRE.

In combination, these findings highlight that localised current density recordings via TCRE produce substantially different signals compared to conventionally acquired electrical potential differences between more widely spaced EEG electrode recordings. Thus, conventional sleep staging criteria should not be applied to TCRE without careful consideration of their temporospatial recording differences. However, attempts to adapt traditional manual scoring criteria to suit TCRE recordings would be difficult and unlikely to be as informative as more comprehensive electrophysiological assessments of temporospatial features available through TCRE recordings.

Future work could consider looking at specific sleep features such as K‐complexes and sleep spindles to further test for more localised activity in TCRE versus EEG. Additional potential applications for TCRE could be to simplify sleep and wake assessments of brain activity with substantially fewer electrodes compared to more labour and computationally intensive source localisation assessments from high‐density EEG recordings. Future research with TCRE could also be useful to further explore features of regional brain activity changes during sleep potentially predictive of important outcomes, such as has been found for memory consolidation (Muehlroth et al., 2019), post‐traumatic stress disorder (Wang et al., 2020), and mortality (Lechat et al., 2022).

Fewer epochs contaminated by artefacts were excluded for TCRE sites in comparison to EEG. TCRE sites exhibited significantly lower beta power, a frequency range commonly associated with extraneous factors such as muscle movement and artefacts, as compared to EEG sites. These findings suggest that, overall, TCRE signals were less contaminated by artefact than EEG signals. Notably, this trend was most pronounced in central and occipital sites, albeit with a small reversal observed for FpZ and Fz, potentially attributable to participants head position while sleeping. TCRE may be particularly advantageous to collect brain activity in studies where artefacts are particularly problematic, such as with samples where muscle artefact is more common (e.g., people with epilepsy [Besio et al., 2013; Besio et al., 2014; Makeyev et al., 2013]) or during wake recordings (e.g., daytime cognition studies [Maess et al., 2016; Moosmann et al., 2009]). Muscle artefacts are most prominent during wakefulness, but may also arise during sleep from arousals, bruxism, or abnormal movements (e.g., REM sleep behaviour disorder), and TCRE may be advantageous in these contexts too. Importantly, conventional noise reduction methods tailored to mitigate EMG artefacts can inadvertently eliminate useful signal features (Fatourechi et al., 2007; Goncharova et al., 2003; Zhu et al., 2022). Thus, TCRE could be a useful approach towards identifying novel markers of sleep and wake that are more problematic with conventional EEG approaches.

While this study provides new insights into TCRE compared to EEG signals during normal sleep in a laboratory, there are some notable limitations. One of the main limitations with TCRE is that it remains unclear how well localised recordings of current density align with traditional recordings of potential differences between more widely spaced electrodes conventionally used to record and define EEG features and sleep stages. For example, given the more focal nature of the recordings, TCRE may well be less sensitive to EEG defined features, such as corticothalamic sleep spindles, partly used to define N2 sleep. On the other hand, TCRE may be more sensitive to more localised electrophysiological features of sleep less evident in conventional EEG recordings. Thus, the ability to characterise localised changes in brain activity during sleep in TCRE versus EEG represents a significant opportunity for further exploration of novel electrophysiological markers that could potentially be more predictive of sleep quality and health outcomes in future research. Thus, although there are visual similarities with conventional EEG, it is unclear if TCRE could be visually scored to approximate more conventional AASM sleep stages. One potential solution could be to score localised sleep. Furthermore, it is unclear whether novel sleep disruption metrics developed using EEG (Lechat et al., 2021), which have been shown to be useful in assessing the impact of sleep disruption on health, would also translate to TCRE signals. Hence, further validation of these metrics is warranted to assess their potential applicability with this electrode system. Due to amplifier channel limits, only 20 TCRE sites could be recorded simultaneously. Thus, the assessment of localised TCRE signal activation is somewhat limited. On the other hand, given that a single TCRE channel approximates a Laplacian transform of 128 conventional EEG electrodes, 20 channels may be more than sufficient to examine both global and localised sleep effects in many settings, particularly when localised areas of interest can be identified (Liu et al., 2020). The outer rings of each TCRE are also readily assessable to support combination recordings of both conventional EEG and TCRE. Hence, TCRE can be readily combined with conventional EEG to allow for sleep assessments and more focussed assessments on localised brain regions. Additionally, the spatial power‐spectral density analyses in this study were largely exploratory, so further research is warranted to better characterise how localised TCRE signals compare to high‐density EEG and location‐specific event‐related potentials. Finally, the present study utilised a group of healthy sleepers. Consequently, the findings cannot be generalised to people with sleep disorders.

Overall, the present findings demonstrate quite marked differences between TCRE and EEG signals collected during sleep in healthy volunteers. Lower relative power was observed in higher frequencies and more relative power in lower frequencies with TCRE compared to EEG electrodes. There was also evidence for more apparent spatiotemporal differences in TCRE versus EEG, with more variable activity across recording sites in TCRE. TCRE also appeared to contain less EMG and movement artefact, particularly in central and occipital electrode locations, resulting in fewer epochs being classed as noisy by automated noise detection and removal analysis. These features suggest that TCRE may be particularly useful for recording localised brain activity during sleep and to obtain recordings potentially less impacted by movement artefacts. These observations clearly warrant further exploration in experimental paradigms better suited to identify localised brain activity, such as in sleep restriction or deprivation, and movement effects such as in a range of sleep disorders, including restless legs syndrome (Willis–Ekbom disease), periodic limb movement disorder, and rhythmic movement disorders (Silber, 2013). TCRE therefore shows promise for capturing brain activity across a wide range of research and clinical applications towards improved understanding of brain activity changes during sleep.

## AUTHOR CONTRIBUTIONS

**Nicole Stuart:** Conceptualization; investigation; writing – original draft; methodology; validation; resources; data curation; software; formal analysis; project administration; writing – review and editing; visualization. **Jack Manners:** Conceptualization; investigation; methodology; writing – original draft; software; writing – review and editing; formal analysis; project administration; data curation; resources. **Eva Kemps:** Writing – review and editing; supervision; methodology; conceptualization. **Duc Phuc Nguyen:** Writing – review and editing; software; formal analysis; data curation. **Bastien Lechat:** Conceptualization; methodology; software; formal analysis; data curation; writing – review and editing. **Peter Catcheside:** Conceptualization; methodology; resources; writing – review and editing; funding acquisition; supervision. **Hannah Scott:** Conceptualization; methodology; investigation; resources; visualization; supervision; writing – review and editing; funding acquisition.

## CONFLICT OF INTEREST

None declared.

## Supporting information


**Data S1.** Supporting information.
**Table S1.** Pairwise comparisons for EEG versus TCRE relative spectral power by power band.
**Figure S1.** EEG trace layout (left) and TCRE trace layout (right) during part of an epoch of wake.
**Figure S2.** EEG trace layout (left) and TCRE trace layout (right) during part of an epoch of N1 sleep.
**Figure S3.** EEG trace layout (left) and TCRE trace layout (right) during part of an epoch of N3 sleep.
**Figure S4.** EEG trace layout (left) and TCRE trace layout (right) during part of an epoch of REM sleep.
**Figure S5.** EEG trace layout (left) and TCRE trace layout (right) during part of an epoch of N3 sleep with a scored arousal.

## Data Availability

The data that support the findings of this study are available from the corresponding author upon reasonable request.
