# Supplementary material for: Tripolar concentric ring electrodes for capturing localised electroencephalography signals during sleep
Source: J Sleep Res. 2024 Mar 27;33(6):e14203. doi: 10.1111/jsr.14203 (PMC11597005; doi:10.1111/jsr.14203)
Supplement: Supplementary file 1 — Data S1. Supporting information. Table S1. Pairwise comparisons for EEG versus TCRE relative spectral power by power band. Figure S1. EEG trace layout (left) and TCRE trace layout (right) during part of an epoch of wake. Figure S2. EEG trace layout (left) and TCRE trace layout (right) during part of an epoch of N1 sleep. Figure S3. EEG trace layout (left) and TCRE trace layout (right) during part of an epoch of N3 sleep. Figure S4. EEG trace layout (left) and TCRE trace layout (right) during part of an epoch of REM sleep. Figure S5. EEG trace layout (left) and TCRE trace layout (right) during part of an epoch of N3 sleep with a scored arousal. [file JSR-33-e14203-s001.docx]

**Figure S1.**

*EEG trace layout (left) and TCRE trace layout (right) during part of an epoch of wake*

**TCRE**

**EEG**


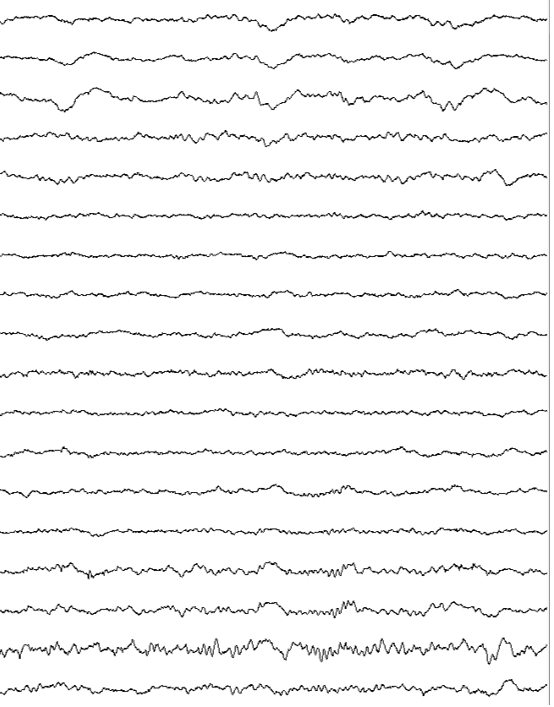

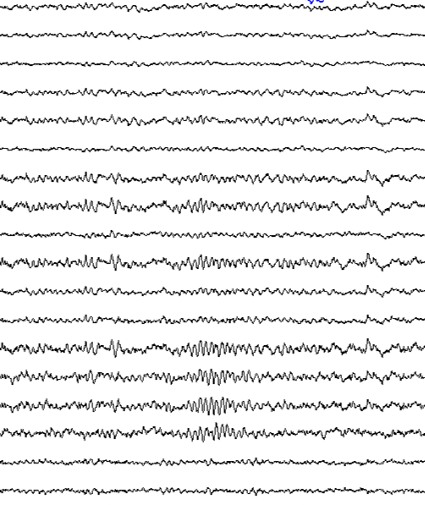


**Fp1**

**FpZ**

**Fp2**

**F3**

**Fz**

**F4**

**C3**

**Cz**

**C4**

**CpZ**

**C5**

**T7**

**Pz**

**O1**

**Oz**

**O2**

**M2**

**M1**

**Figure S2.**

*EEG trace layout (left) and TCRE trace layout (right) during part of an epoch of N1 sleep*

**TCRE**

**EEG**


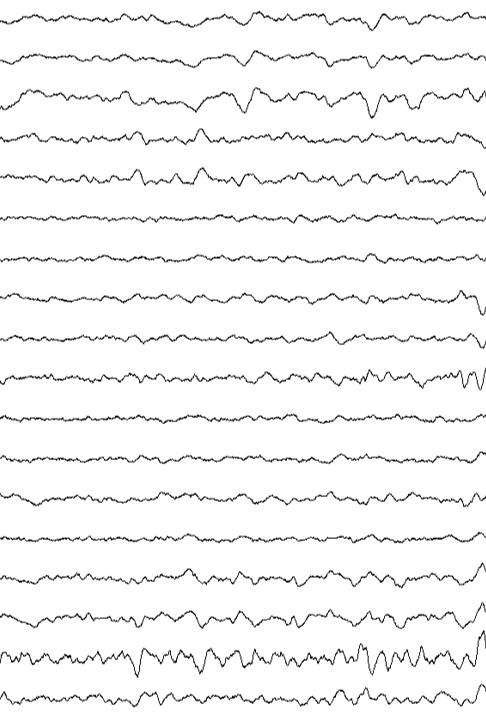

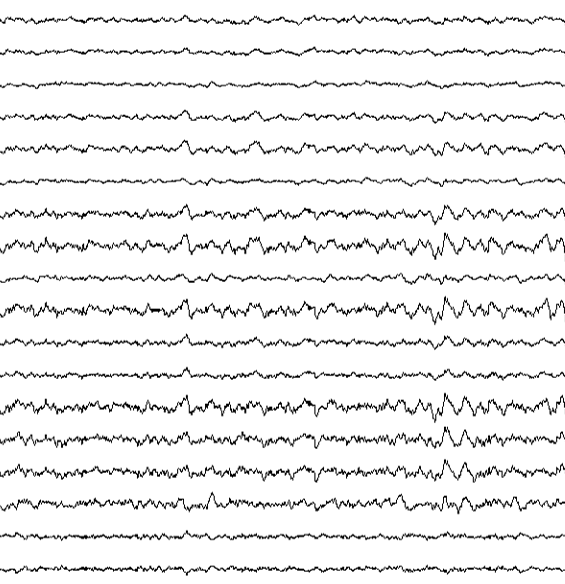


**Fp1**

**FpZ**

**Fp2**

**F3**

**Fz**

**F4**

**C3**

**Cz**

**C4**

**CpZ**

**C5**

**T7**

**Pz**

**O1**

**Oz**

**O2**

**M2**

**M1**

**Figure S3.**

*EEG trace layout (left) and TCRE trace layout (right) during part of an epoch of N3 sleep*

**EEG**


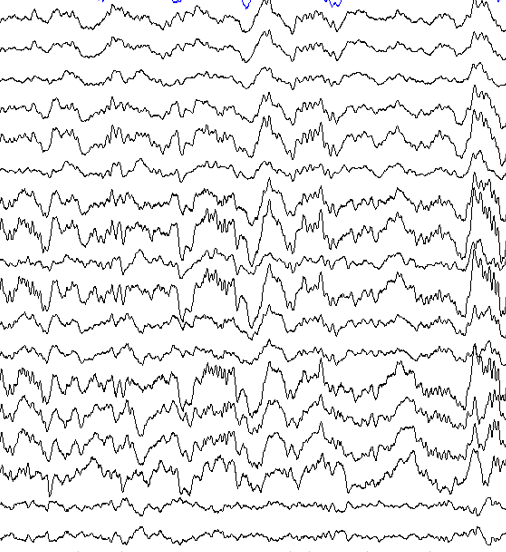


**TCRE**


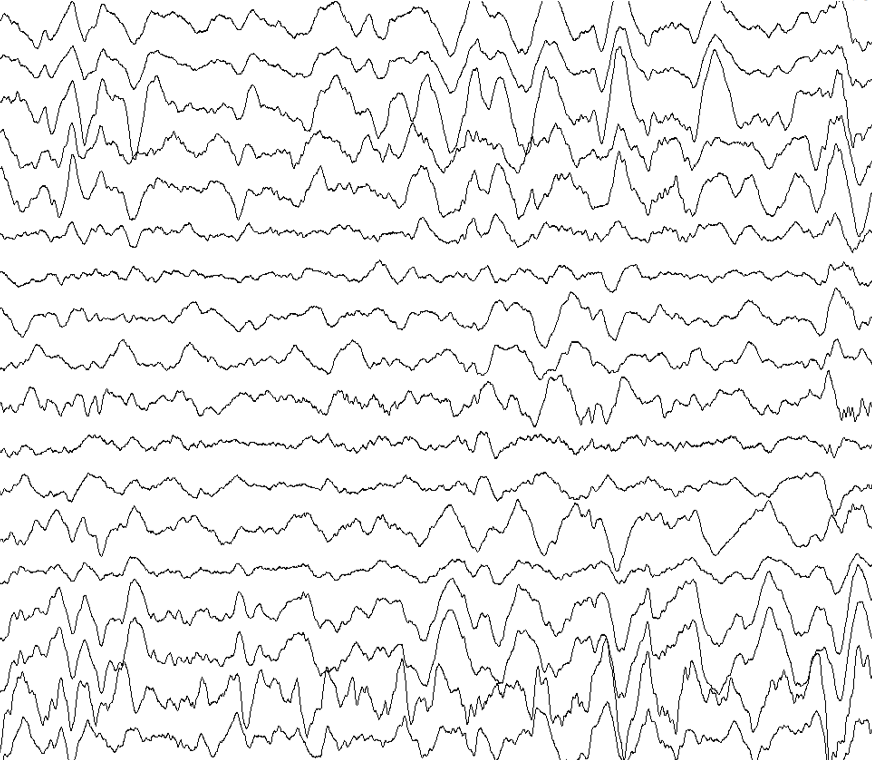


**Fp1**

**FpZ**

**Fp2**

**F3**

**Fz**

**F4**

**C3**

**Cz**

**C4**

**CpZ**

**C5**

**T7**

**Pz**

**O1**

**Oz**

**O2**

**M2**

**M1**

**Figure S4.**

*EEG trace layout (left) and TCRE trace layout (right) during part of an epoch of REM sleep*

**TCRE**

**EEG**


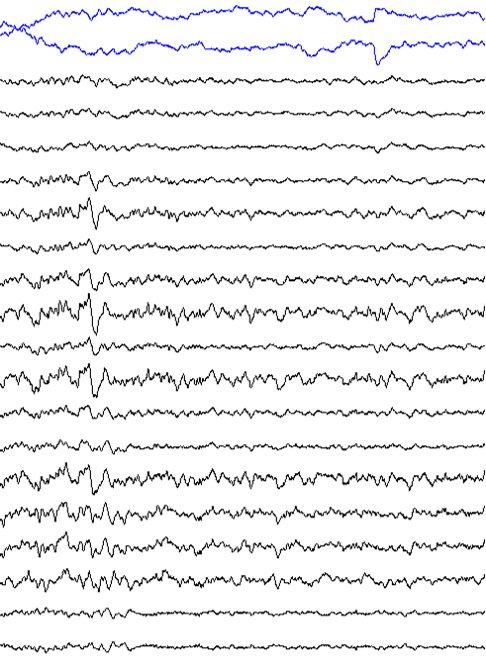


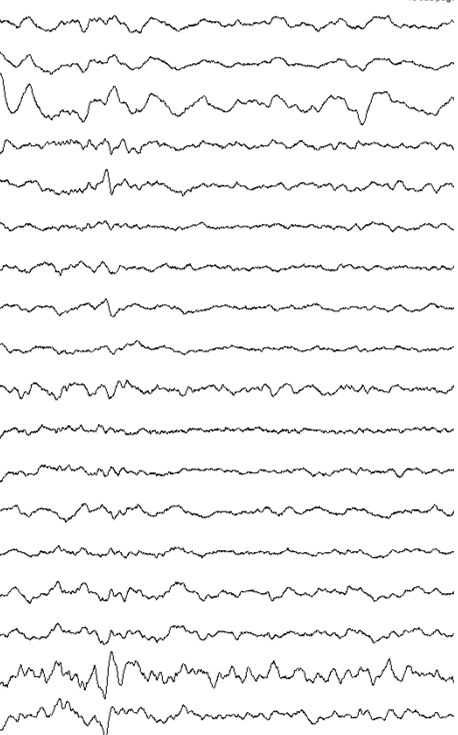


**Fp1**

**FpZ**

**Fp2**

**F3**

**Fz**

**F4**

**C3**

**Cz**

**C4**

**CpZ**

**C5**

**T7**

**Pz**

**O1**

**Oz**

**O2**

**M2**

**M1**

**Figure S5.**

*EEG trace layout (left) and TCRE trace layout (right) during part of an epoch of N3 sleep with a scored arousal*

**TCRE**

**EEG**


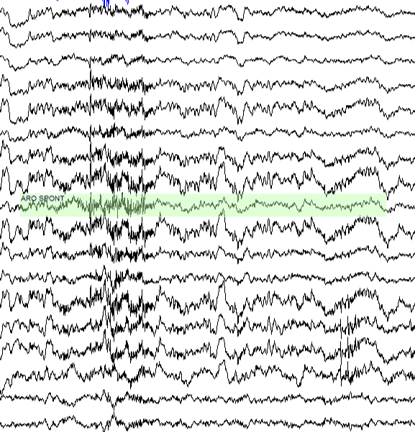


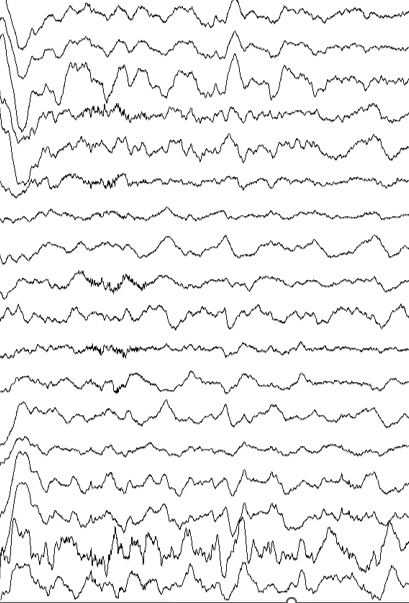


**Fp1**

**FpZ**

**Fp2**

**F3**

**Fz**

**F4**

**C3**

**Cz**

**C4**

**CpZ**

**C5**

**T7**

**Pz**

**O1**

**Oz**

**O2**

**M2**

**M1**

**Table S1.**

*Pairwise comparisons for EEG vs TCRE relative spectral power by Power Band*

| **Power Band** | **Stage** | **EEG**  **(Mean±SE)** | **TCRE**  **(Mean±SE)** | **Mean Difference (EEG-TCREMean 95%CI)** | **Sig.**  ***p*** | **Cohen’s**  ***d*** |
| --- | --- | --- | --- | --- | --- | --- |
| Alpha | Wake | .292 (.005) | .108 (.005) | .185 (0.177, 0.192) | < .001 | 8.27 |
|  | N1 | .151 (.005) | .045 (.005) | .107 (0.099, 0.114) | < .001 | 4.79 |
|  | N2 | .129 (.005) | .038 (.005) | .091 (0.084, 0.099) | < .001 | 4.07 |
|  | N3 | .060 (.006) | .021 (.006) | .039 (0.031, 0.047) | < .001 | 1.49 |
|  | REM | .159 (.005) | .046 (.005) | .113 (0.105, 0.120) | < .001 | 5.05 |
| Beta | Wake | .175 (.003) | .037 (.003) | .138 (0.132, 0.143) | < .001 | 10.29 |
|  | N1 | .085 (.003) | .027 (.003) | .058 (0.053, 0.063) | < .001 | 4.32 |
|  | N2 | .042 (.003) | .019 (.003) | .023 (0.018, 0.029) | < .001 | 1.71 |
|  | N3 | .012 (.003) | .008 (.003) | .004 (-0.002, 0.009) | 0.199 | .31 |
|  | REM | .073 (.003) | .028 (.003) | .045 (0.039, 0.050) | < .001 | 3.35 |
| Delta | Wake | .372 (.009) | .772 (.009) | -.4 (-0.410, -0.389) | < .001 | 9.94 |
|  | N1 | .553 (.009) | .847 (.009) | -.293 (-0.304, -0.283) | < .001 | 7.28 |
|  | N2 | .637 (.009) | .872 (.009) | -.235 (-0.246, -0.224) | < .001 | 5.84 |
|  | N3 | .838 (.010) | .929 (.010) | -.091 (-0.102, -0.080) | < .001 | 2.09 |
|  | REM | .561 (.009) | .842 (.009) | -.282 (-0.292, -0.271) | < .001 | 7.01 |
| Sigma | Wake | .057 (.001) | .017 (.001) | .041 (0.039, 0.043) | < .001 | 9.17 |
|  | N1 | .054 (.001) | .013 (.001) | .041 (0.039, 0.043) | < .001 | 9.17 |
|  | N2 | .066 (.001) | .014 (.001) | .053 (0.050, 0.055) | < .001 | 11.85 |
|  | N3 | .018 (.001) | .006 (.001) | .013 (0.010,0.015) | < .001 | 2.98 |
|  | REM | .043 (.001) | .012 (.001) | .031 (0.029,0.033) | < .001 | 6.93 |
| Theta | Wake | .103 (.003) | .067 (.003) | .036 (0.032, 0.041) | < .001 | 2.68 |
|  | N1 | .157 (.003) | .069 (.003) | .088 (0.083, 0.093) | < .001 | 6.56 |
|  | N2 | .126 (.003) | .058 (.003) | .068 (0.063, 0.073) | < .001 | 5.07 |
|  | N3 | .071 (.003) | .036 (.003) | .036 (0.031, 0.041) | < .001 | 2.75 |
|  | REM | .165 (.003) | .071 (.003) | .093 (0.088, 0.098) | < .001 | 6.93 |

*Note*. CI = 95%
